# Three novel *MTM1* pathogenic variants identified in Japanese patients with X‐linked myotubular myopathy

**DOI:** 10.1002/mgg3.621

**Published:** 2019-03-18

**Authors:** Atsuko Nishikawa, Aritoshi Iida, Shinichiro Hayashi, Mariko Okubo, Yasushi Oya, Gaku Yamanaka, Ikuko Takahashi, Ikuya Nonaka, Satoru Noguchi, Ichizo Nishino

**Affiliations:** ^1^ Department of Neuromuscular Research National Institute of Neuroscience, National Center of Neurology and Psychiatry Tokyo Japan; ^2^ Department of Clinical Genome Analysis Medical Genome Center, National Center of Neurology and Psychiatry Tokyo Japan; ^3^ Department of Neurology National Center Hospital, National Center of Neurology and Psychiatry Tokyo Japan; ^4^ Department of Pediatrics Tokyo Medical University Hospital Tokyo Japan; ^5^ Department of Pediatrics Akita University Faculty of Medicine Akita Japan

**Keywords:** *MTM1*, novel variants, targeted gene panel system, X‐linked myotubular myopathy

## Abstract

**Background:**

X‐linked myotubular myopathy (XLMTM) is a form of the severest congenital muscle diseases characterized by marked muscle weakness, hypotonia, and feeding and breathing difficulties in male infants. It is caused by mutations in the myotubularin gene (*MTM1*).

**Methods:**

Evaluation of clinical history and examination of muscle pathology of three patients and comprehensive genome analysis on our original targeted gene panel system for muscular diseases.

**Results:**

We report three patients, each of whom presents distinct muscle pathological features. The three patients have novel hemizygous *MTM1* variants, including c.527A>G (p.Gln176Arg), c.595C>G (p.Pro199Ala), or c.688T>C (p.Trp230Arg).

**Conclusions:**

All variants were assessed as “Class 4 (likely pathogenic)” on the basis of the guideline of American College of Medical Genetics and Genomics. These distinct pathological features among the patients with variants in the second cluster of PTP domain in *MTM1* provides an insight into microheterogeneities in disease phenotypes in XLMTM.

## 1 INTRODUCTION

X‐linked myotubular myopathy (XLMTM:OMIM #310400) is among the most severe congenital muscle diseases characterized by marked muscle weakness, hypotonia, and feeding and breathing difficulties in male infants (Sarnat, Roth, & Jimenez, 1981; Spiro, Shy, & Gonatas, 1966). Typically, most of XLMTM patients die during the first year of life due to respiratory insufficiency (Wallgren‐Pettersson et al., 1995). Muscle biopsy shows characteristic histopathologic features, with small rounded myofibers that are predominantly type 1, centrally located myonuclei, and accumulations of mitochondria in peripheral halos (Abath Neto et al., 2016). The estimated incidence of XLMTM is 1 in 50,000 live male births (Jungbluth, Wallgren‐Pettersson, & Laporte, 2008). So far more than 290 different mutations have been identified in the causative gene for XLMTM, *MTM1* (OMIM: #300415; NM_000252.2; Laporte et al., 1996; HGMD Professional 2018.1). From the phenotypes of *Mtm1*‐null mice, it has been suggested that *MTM1* should be essential for skeletal muscle maintenance rather than muscle development (Buj‐Bello et al., 2002).

Myotubularin, the gene product of *MTM1*, is a 3‐phosphatase for phosphatidylinositol 3‐monophosphate and phosphatidylinositol 3,5‐bisphosphates (Hnia, Vaccari, Bolino, & Laporte, 2012). Myotubularin has been implicated in several cellular processes including endocytosis, membrane trafficking, cell proliferation and differentiation, survival, autophagy, cytoskeleton, and cell junction dynamics (Hnia et al., 2011; Noguchi, Fujita, Murayama, Kurokawa, & Nishino, 2005). It has been demonstrated that abnormal T‐tubule morphology and sarcoplasmic reticulum dilation lead to disruption of excitation‐contraction coupling and subsequent defects in calcium regulation and muscle contraction in XLMTM (Dowling et al., 2009). Recent studies also indicated that myotubularin regulates DNM2 expression via BIN1, both of which are the products of the genes causative of centronuclear myopathy (Cowling et al., 2014). In this study, we identified three novel variants in *MTM1* in infantile‐onset XLMTM patients.

## 2 METHODS

### 2.1 Targeted gene panel sequencing (or mutation analysis)

Patients 1 and 2 were diagnosed by Ion PGM sequencer (Thermo Fisher Scientific, MA) in combination with the original targeted gene panel containing 41 known causative genes for congenital myopathy (Nishikawa, Mitsuhashi, Miyata, & Nishino, 2017). We also screened *MTM1* pathogenic variant(s) in patient 3 by direct sequencing of the entire coding exons and their flanking regions.

### 2.2 Ethic compliance

All clinical information and materials from the patients were obtained for diagnostic purpose and permitted for scientific use with written informed consent. The study was approved by the Ethical Committee of National Center of Neurology and Psychiatry.

## 3 RESULTS

### 3.1 Case reports

Patient 1 is a 5‐year and 1‐month‐old Japanese boy. He was suffering from severe neonatal asphyxia which required mechanical ventilation since birth. Tracheostomy and gastrostomy was performed at age 7 months and 4 years, respectively. He had high‐arched palate and generalized muscle weakness including facial muscles. He had acquired no head control but could sit independently at age 5 years.

Patient 2 is a 5‐month‐old Japanese boy. He presented with severe neonatal asphyxia. Although respiratory failure appeared soon after birth and intubation was required, he was extubated at age 7 days. He had generalized muscle weakness and did not show head control. There were no abnormalities in brain MRI, G‐band karyotyping analysis, and Prader‐Willi FISH test.

Patient 3 is an 18‐year‐old Japanese man. He was markedly hypotonic at birth and needed oxygen inhalation until 3 weeks of age. He had no difficulty in sucking and swallowing. Regardless of predominantly proximal muscle weakness, he eventually acquired head control, stood up and walked independently. At age 17 years, noninvasive positive airway pressure ventilation was started due to hypercapnia, albeit he was still ambulant. Based on Herman et al. (Herman, Finegold, Zhao, Gouyon, & Metzenberg, 1999), the phenotypes of Patients 1 and 2 were classified into “severe,” and those of Patient 3 were “moderate.”

**Figure 1:**
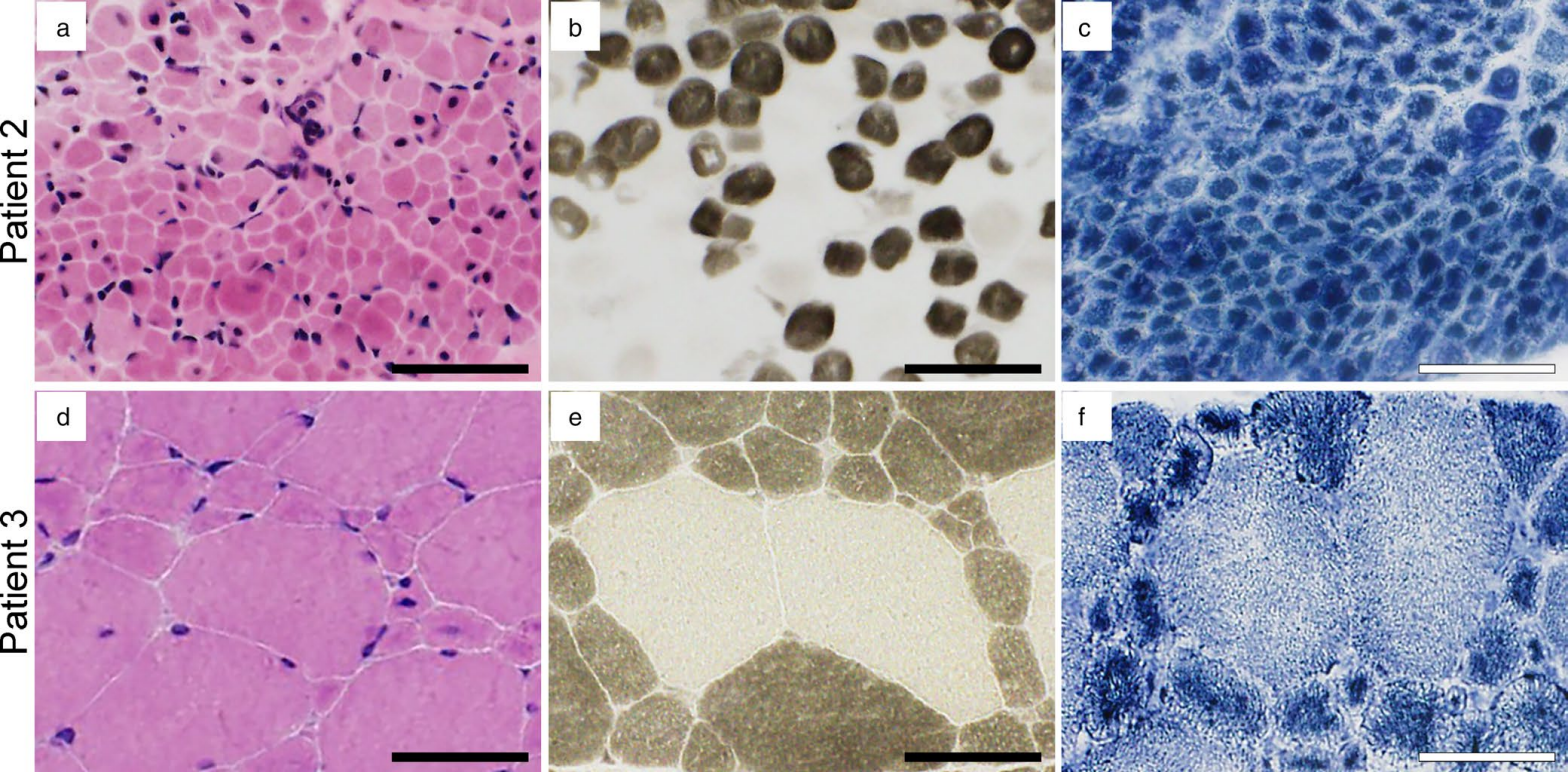
Histological analyses of the muscle biopsies from patients. (a and d) Hematoxylin and eosin staining; (b and e) Myosin ATPase (pH10.7); (c and f) Nicotinamide adenine dinucleotide tetrazolium reductase staining. Bar: 50 µm

None of the patients showed abnormalities in brain MRI and blood/urine biochemical examinations. Serum creatine kinase levels were within normal range in all patients. On muscle pathology in patients 1 (at age 5 years and 1 month) and 2 (at age 5 months), almost all fibers were round in shape and small in size and had peripheral halo and centrally placed nuclei (Figure 1a–c). In contrast, muscle pathology in patient 3 (at age 6 years) showed bimodal fiber size distribution with larger type 2 fibers and smaller clustered type 1 fibers (Figure 1d–f). Type 1 fiber predominance and atrophy was seen in patients 1 and 3, and type 2B fiber deficiency in patients 2 and 3 (data not shown).

### 3.2 Genetic analysis

**Figure 2:**
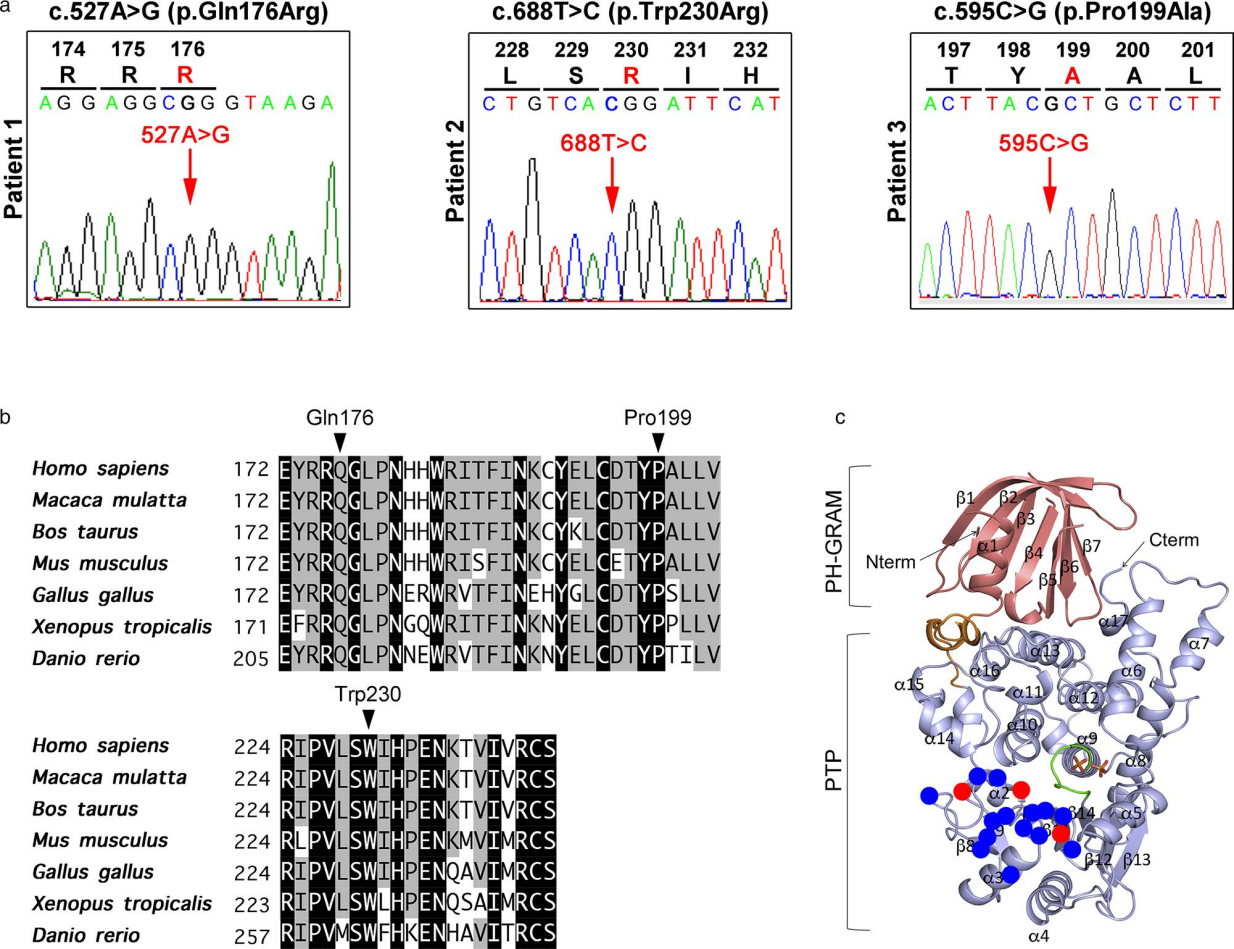
(a) Three novel hemizygous variants identified in this study (c.527A>G, c.688T>C, and c.595C>G). (b) Multiple alignment of the myotubularin amino acid sequences among different species. The variant sites (Gln176, Pro199, and Trp230) are highly conserved throughout evolution. (c) The variant sites in XLMTM on predicted steric structure of myotubularin protein based on the crystal structure of MTMR1. The crystal structure was obtained from Bong et al. (2016). The variant sites (Gln176, Pro199, and Trp230) and those of previously reported are indicated in red and blue, respectively

Hemizygous variants, c.527A>G (p.Gln176Arg) and c.688T>C (p.Trp230Arg) were identified in patients 1 and 2, respectively (Figure 2a). Both variants were confirmed by Sanger sequencing. In patient 3, a c.595C>G (p.Pro199Ala) in exon 8 was found (Figure 2a). All of variants were predicted to be nonsynonymous amino acid substitutions. The mutated amino acid residues were localized in the protein tyrosine phosphatase (PTP) domain and were evolutionally conserved from human to zebrafish (Figure 2b). At least two of three in silico mutation prediction tools (SIFT, PolyPhen2 and Mutation Taster) showed the variants as “deleterious” (Table S1).

The identified three variants were not deposited in any databases, including dbSNP, Exome Aggregation Consortium, Human Gene Mutation Database, Human Genetic Variation Database. The variant, c.688T>C is present in ClinVar due to be deposited by the EGL Genetic Diagnostics, Eurofins Clinical Diagnostics and the Genetic Services Laboratory, University of Chicago. However, Clinical significance of this variant in ClinVar has been represented as “Conflicting interpretations of pathogenicity (18 March 2013).” The other two variants were not deposited in ClinVar (as the end of April 2018). Finally, we evaluated the variants with the guideline for sequence variants in American College of Medical Genetics and Genomics (ACMG) (Richards et al., 2015). Three variants are considered to be “Class 4 (likely pathogenic)” (Table S2).

## 4 DISCUSSION

In this study, we report three patients with novel variants in *MTM1*. First we evaluated and classified these variants with the guideline in ACMG (Richards et al., 2015) (Table S2).

1. The c.688T>C (p.Trp230Arg) is a same amino acid substitution as previously established pathogenic variant, c.688T>A (p.Trp230Arg) (Tsai et al., 2005).
2. All variants are located on PTP domain in MTM1 (Begley et al., 2003). The variants fulfill the category PM1 (Mutational hotspot and/or critical and well‐established functional domain).
3. All variants are absent from controls in any databases, including dbSNP, Exome Aggregation Consortium, Human Gene Mutation Database, Human Genetic Variation Database. The two variants, c.527A>G and c.595C>G, were not deposited in ClinVar. However, the variant, c.688T>C is present in ClinVar as “Conflicting interpretations of pathogenicity by the EGL Genetic Diagnostics, Eurofins Clinical Diagnostics and the Genetic Services Laboratory, University of Chicago (Category PM2: Absent from controls). In this study, we described the correlation between genotypes and phenotypes.
4. Different pathogenic amino acid substitutions in each of the three codons were reported (Category PM5: different substitutions in same codon have reported).
5. All variants are predicted as “deleterious” by multiple lines of computational tools. The affected amino acids were evolutionally conserved among different species (Category PP3: computational evidence).
6. Patient's phenotypes are highly specific for XMTM1 based on clinical and pathological examinations (Category PP4: highly specific phenotypes for a disease).

The variants, c.688T>C (p.Trp230Arg) and c.595C>G (p.Pro199Ala) are satisfied the categories, PS1, PM2, PM5, PP3, PP4, and PM1, PM2, PM5, PP3 PP4, respectively. Hence, these variants categorized Class 4 (likely pathogenic). The variant, c.527A>G (p.Gln176Arg) is also satisfied PM2, PM5, PM3, and PM4. The variant also categorized Class 4 (likely pathogenic).

Patients 1 and 2 had typical clinicopathological manifestations while patient 3 showed much milder features, such as independent walking and unassisted breathing, in addition to bimodal fiber size distribution with centronucleated small type 1 fibers. We have previously reported similar milder cases (Tsai et al., 2005). Recently, we also reported pathological features of skeletal muscles from XLMTM fetus, which showed normal myofibrillar alignment without “myotubular” appearance (Hamanaka et al., 2016). These data again suggest that myotubularin plays important roles in maintaining normal myofibrillar alignment in matured muscle fibers and its defects result in “myotubular” phenotype. These roles of myotubularin may be severely affected by the variants, c.527A>G (p.Gln176Arg) and c.688T>C (p.Trp230Arg) identified in patients 1 and 2, while only partially affected by c.595C>G (p.Pro199Ala) in patient 3. The large cohort study to associate among the genetic mutation, clinical manifestation, and pathological features may help deducing conclusive genotype‐phenotype correlation among XLMTM patients.

Based on crystal structures of MTMR1 and MTMR2, and the identity of primary sequences among MTMR1, MTMR2, and myotubularin, the structural influences of the mutated residues in myotubularin identified in XLMTM have been discussed (Begley et al., 2003; Bong et al., 2016). The majority of residues at the sites of missense mutations has been reported to be buried in the hydrophobic core in the PTP domain (Figure 2c). There are two clusters of mutation sites on myotubularin sequence. Residues at mutation sites in the first cluster at 346‐421 participate to form substrate binding pocket, while residues at mutation sites in the second cluster at 179‐241 serve a solvent‐accessible/surface‐exposure region outside of minimal structural core of PTP domain. The mutations located in the second cluster opposite to the substrate binding site, have been suggested to indirectly affect the enzymatic activity via their disturbed hydrogen‐bond interaction with residues forming a core of the PTP or to induce the instability of myotubularin protein. In fact, it has been reported that the substitutions, Arg to Gly at 184, Pro to Leu at 205, Arg to Leu at 241 in the second cluster severely compromised catalytic activity of myotubularin (Begley et al., 2003). All of our identified mutations in this study are located at the second cluster and predicted to be surface‐exposed residues. These variants may also indirectly affect the enzymatic activity. The detailed effects of the variants on structural change and enzymatic activity of myotubularin molecule should be clarified for considering pathogenesis and phenotype‐genotype correlation in XLMTM.

## CONFLICT OF INTEREST

The authors declare no conflict of interest.
