# Supplementary material for: Three novel MTM1 pathogenic variants identified in Japanese patients with X‐linked myotubular myopathy
Source: Mol Genet Genomic Med. 2019 Mar 18;7(5):e621. doi: 10.1002/mgg3.621 (PMC6503166; doi:10.1002/mgg3.621)
Supplement: Supplementary file 1 [file MGG3-7-e621-s001.docx]

Supplementary Table 1. Evaluation of three *MTM1* missense mutations with three mutation prediction tools

|  | SIFT  (Score) | PolyPhen-2 (Score) | Mutation Taster (Score) |
| --- | --- | --- | --- |
| c.527A>G (p.Gln176Arg) | Tolerated (0.17) | Possibly damaging (0.95) | Disease causing (1) |
| c.595C>G (p.Pro199Ala) | Damaging (0) | Probably damaging (1) | Disease causing (1) |
| c.688T>C (p.Trp230Arg) | Damaging (0) | Probably damaging (1) | Disease causing (1) |
