# Supplementary material for: Three novel MTM1 pathogenic variants identified in Japanese patients with X‐linked myotubular myopathy
Source: Mol Genet Genomic Med. 2019 Mar 18;7(5):e621. doi: 10.1002/mgg3.621 (PMC6503166; doi:10.1002/mgg3.621)
Supplement: Supplementary file 2 [file MGG3-7-e621-s002.docx]

Supplementary table 2. Assessment of three variants by the guideline in ACMG

| Variant | PS1 | PS2 | PS3 | PS4 | PM1 | PM2 | PM3 | PM4 | PM5 | PM6 | PP1 | PP2 | PP3 | PP4 | PP5 |
| --- | --- | --- | --- | --- | --- | --- | --- | --- | --- | --- | --- | --- | --- | --- | --- |
| c.527A>G (p.Gln176Arg) | ⎯ | ⎯ | ⎯ | ⎯ | Yes | Yes | ⎯ | ⎯ | Yes | ⎯ | ⎯ | ⎯ | Yes | Yes | ⎯ |
| c.688T>C (p.Trp230Arg) | Yes | ⎯ | ⎯ | ⎯ |  | Yes | ⎯ | ⎯ | Yes | ⎯ | ⎯ | ⎯ | Yes | Yes | ⎯ |
| c.595C>G (p.Pro199Ala) | ⎯ | ⎯ | ⎯ | ⎯ | Yes | Yes | ⎯ | ⎯ | Yes | ⎯ | ⎯ | ⎯ | Yes | Yes | ⎯ |

⎯: the variant did not fulfill criteria
